# Pediatric Health at the Crossroads of Climate Change, Food Insecurity, and Malnutrition

**DOI:** 10.1016/j.advnut.2026.100658

**Published:** 2026-05-14

**Authors:** Tindara Scirocco, Daniela Martini, Carlo Agostoni, Mario C.B. Raviglione, Stefano Bocchi, Cristiana Berti

**Affiliations:** 1 Department of Life Sciences and Public Health, Università Cattolica del Sacro Cuore, Rome, Italy; 2 Division of Human Nutrition, Department of Food, Environmental and Nutritional Sciences, University of Milan, Milan, Italy; 3 Pediatric Unit, Fondazione IRCCS Ca’ Granda Ospedale Maggiore Policlinico, Milan, Italy; 4 Department of Clinical Sciences and Community Health, University of Milan, Milan, Italy; 5 Centre for Multidisciplinary Research for Global Health (MAGH), University of Milan, Milan, Italy; 6 Department of Environmental Science and Policy, University of Milan, Milan, Italy

**Keywords:** climate change, food and nutrition insecurity, infectious diseases, noncommunicable diseases, malnutrition, pediatric populations, agroecology, eco-agrofood system, breastfeeding, sustainable healthy diet

## Abstract

Climate change (CC) poses a major global threat to the health of current and future generations, disproportionately affecting pediatric populations. Investigating the links between CC and pediatric diseases is crucial to inform research and prevention strategies aimed at breaking the transgenerational cycle of social inequalities. This narrative review explores the complex interactions between early-life exposures to CC, food insecurity, and malnutrition, and their impact on infectious and noncommunicable diseases (NCDs) in pediatric populations. Data reveal a concerning global scenario: half of the world’s children live in areas highly vulnerable to CC; malaria, enteric, and lower respiratory-tract infections account for ∼60% of the global communicable disease burden and related deaths in children and adolescents; over 2.1 billion people under-20 suffer from NCDs; almost 865 million children under-15 experience moderate-to-severe food insecurity; and millions of children under-5 face stunting (150.2), wasting (42.8), or obesity (35.5). The greatest burdens fall on low- and middle-income countries and the most disadvantaged households. Although the causal pathways and mechanisms linking CC to health outcomes have not been fully elucidated, epidemiological evidence shows that exposure from conception through adolescence increases risks of acute and chronic diseases, potentially altering lifelong health trajectories. This is plausibly driven by climate-induced disruptions in eco-agrofood systems, which compromise nutrition security and worsen malnutrition. Food systems are both vulnerable to and significant contributor to CC, and poor dietary patterns further amplify disease burdens. Addressing these intertwined challenges requires a holistic approach promoting healthy, sustainable, and equitable diets from infancy through adolescence, and employing an integrated “glocal” strategy taking into account both global and local contexts. Cross-sector collaboration and targeted pediatric research are paramount to enhance understanding of causal pathways and develop effective interventions to safeguard child health and well-being within a planetary health framework.

**Statement of Significance**

This review critically examines how early-life exposure to climate-related disruptions in eco-agrofood systems exacerbates the pediatric disease burdens. It also provides actionable insights to help guide research, policy, and actions tackling these interrelated challenges, focusing on the connection between climate change and the food environments, from a “glocal” perspective, ultimately protecting child health.

## Introduction

**FIGURE 1:**
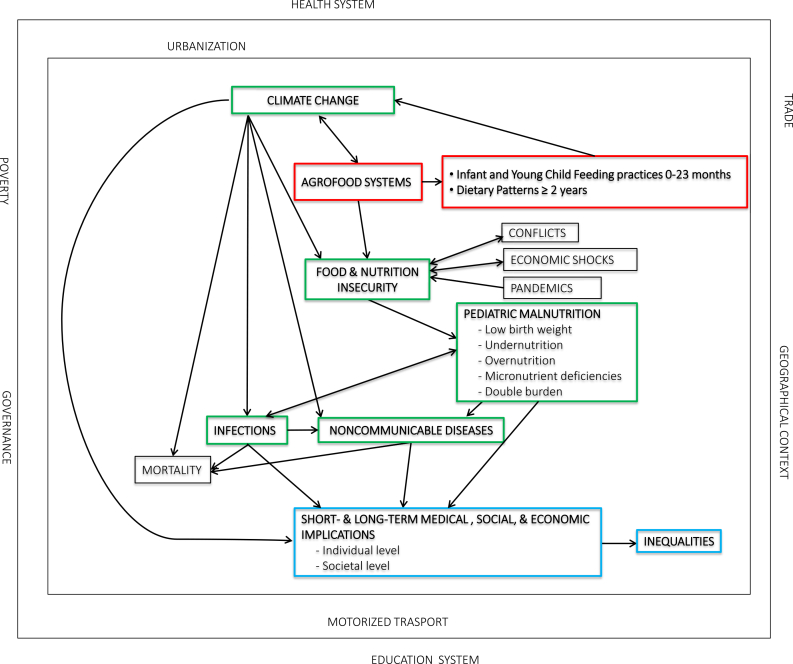
Early exposure to the climate change–agrofood system dyad: interconnected drivers and lifelong health impacts on pediatric populations. Climate change and the agrofood system interact dynamically and bidirectionally, shaping early-life nutrition and lifelong pediatric health. Climate change acts as both a direct and indirect driver of pediatric health, increasing the risk of infections, exacerbating NCDs, and contributing to mortality. Indirectly, climate-driven disruptions of eco-agrofood systems alter food production, availability, and access, influencing infant and young child feeding (0–23 mo) and dietary patterns in children aged ≥2 y. These disruptions drive food and nutrition insecurity, which, combined with stressors such as conflicts, economic shocks, and pandemics, can impact all forms of pediatric malnutrition, as well as predictive risk factors such as low birth weight. Malnutrition, in turn, increases vulnerability to infections and early-onset of NCDs, with long-term health, social, and economic consequences that reinforce cycles of inequality. These pathways are embedded within broader structural and contextual factors, including urbanization, poverty, governance, trade, geography, motorized transport, and education, which shape exposure, vulnerability, and resilience across the life course. Directionality and feedback loops highlight the complex, cumulative, and interconnected nature of these systems. NCDs, noncommunicable diseases.

Climate change (CC) is increasingly recognized as a major global health emergency, jeopardizing human lives and the foundations of health and well-being, with damaging consequences that go beyond immediate risks, therefore impacting today and future generations [1]. CC is already influencing the epidemiology of infectious, cardiovascular, respiratory, and mental diseases, with deep consequences for public health. Nearly half of the world’s children live in areas highly susceptible to CC [2]. According to projections from *The State of the World’s Children 2024*, children’s exposure to climate hazards is expected to rise markedly by the 2050s compared with the 2000s [3]. If current trends continue, the number is projected to rise 8-fold for extreme heatwaves, 3.1-fold for river floods, 1.7-fold for wildfires, 1.3-fold for droughts, and 1.2-fold for tropical cyclones [3]. Low- and middle-income countries (LMICs) are most acutely experiencing the adverse effects of CC, due to their higher exposure to risk factors, the limited access to vulnerable healthcare services, and the overall social deprivation and poverty. Although public awareness of the health implications of CC remains limited, the scientific understanding of these pathways is well established. A substantial and rapidly expanding body of literature, the consolidation of Planetary Health, defined as “the health of human civilizations and the natural systems on which they depend” [4], as a formal discipline, the emergence of dedicated journals, and the reports released by major health organizations all reflect the strong scientific consensus on the links between CC and health [5]. CC impacts mortality, disease incidence or severity, and risk factors, through both direct effects (e.g., extreme temperatures or weather events) and indirect effects, including changes in vector-borne disease patterns, destruction of essential infrastructure and services, food shortages, declining nutrient density, ecosystem disturbances, increased aeroallergens and air pollution, population displacement, and economic shocks [6,7]. Figure 1 schematizes the complex interactions among early exposure to the CC, food insecurity, and malnutrition, highlighting their enduring impacts on pediatric health. It also identifies the main determinants, system drivers, and contextual conditions influencing these pandemics at both individual and global scales.

At the 12th International Conference on Nutrition and Growth held in Athens (Greece) in 2025, the up-to-date knowledge about the interconnectedness among CC, food systems, and diseases was discussed to identify potential solutions for future research and policy recommendations. Studying the potential impacts of climate hazards on prenatal and pediatric health is an emerging area of research, and the existing literature on this topic is still limited, both quantitatively and qualitatively [6,8,9]. Accordingly, this narrative review critically examines relevant findings and key topics related to the food chain and the cascade of implications for infectious diseases and noncommunicable diseases (NCDs) in a changing climate, with a focus on pediatric health. It aims to address 2 core questions: *1*) How may early-life exposures to climate-driven disruptions in food systems affect pediatric health outcomes, particularly the risk for infectious and NCDs? and *2*) What strategies and interventions can help mitigate these impacts and support pediatric health in the context of CC?

## Methods

Figure 1 informed the conceptualization of this narrative review and its methodological strategy. We conducted a narrative review of the literature with an international scope using PubMed, Web of Science, and Google Scholar to identify evidence on the interconnections among climate, health, and nutrition in pediatric populations. Search terms included combinations of keywords related CC, food security, malnutrition, infectious diseases, NCDs, malnutrition, food security, and pediatric populations. We included original studies, systematic reviews, meta-analyses, and reports from international organizations published within the past 10 y. Eligible sources had to address 1 or more of the following: *1*) the effects of climate-driven environmental changes on infectious and NCDs, including pregnancy outcomes as a critical window shaping offspring health; *2*) the impacts of climate-related hazards on agrofood systems; *3*) epidemiological trends in food insecurity, pediatric malnutrition, and child health to contextualize the extent of the overlapping risks faced by children; and *4*) potential strategies to address these interconnected challenges. Additional sources were identified through reference list screening.

Although this narrative review is not exhaustive, it provides an integrated overview of the links between CC, food systems, and pediatric health, and it highlights key leverage points for promoting healthy and sustainable diets.

## CC: Repercussions on Acute and Chronic Diseases in Pediatric Populations

There is a rising body of scientific evidence on the negative impacts of climate-related exposures and child health [6]. Pediatric populations are particularly susceptible to the detrimental effects of CC because of their physiology and social/behavioral needs [10,11]. Their developing organs, tissues, and systems, including the immune system and thermoregulatory function, are not fully mature, making them less protected against climate-related hazards. Their higher metabolic rate relative to body size increases their demand for nutrients, fluids, and air, thereby making them more vulnerable to food and water shortages and to greater exposure to environmental pollutants. They tend to exhibit risk-unaware behaviors and depend on adult care. Furthermore, their capacity to adapt is influenced by contextual conditions such as geographical, political, and socioeconomic factors, and the severity of climate events. Nearly 90% of CC-related disease burden is carried by children [2], and the effects can begin before birth [12]. Any related physiological or metabolic disturbance can affect health and functioning from childhood to adulthood, with a wide range of health and social implications accumulating over time and contributing to an intergenerational cycle of inequities [6,10].

### CC and infectious diseases

Children are disproportionately affected by climate-sensitive infectious diseases, especially due to the immaturity of their immune systems and their higher exposure to environmental hazards [13]. Rising temperature and humidity fluctuations, both accelerated by CC, directly influence pathogen survival, replication, and transmission [14]. Additionally, environmental pollution exacerbated by CC contributes to immune dysregulation through airway inflammation and impaired host defense [15], thereby increasing susceptibility to respiratory infections [6,11,14].

#### Vector-borne diseases

Global warming fosters microbial persistence in the environment, reshaping traditional patterns of disease epidemiology [16]. Global warming has enabled mosquito and tick vectors to expand into temperate regions, such as Europe and North America [6,17,18]. These trends are leading to increased environmental suitability for vector-borne diseases such as malaria, Zika, dengue, West Nile, and Lyme disease, with direct consequences for the pediatric population [17,19]. These changes are supported by robust epidemiological trends. According to the 2024 *Lancet Countdown*, between 1951 to 1960 and 2014 to 2023, the global transmission risk (R_0_) for *Aedes albopictus*, a major vector of dengue, chikungunya, and Zika, increased by 46.3%, reaching 60.4% in high Human Development Index (HDI) countries, including those in Europe [20]. Similarly, the transmission risk for Lyme disease rose by 24.4% globally, with climate suitability for *Ixodes* tick vectors increasing by 65.5% in high-HDI regions such as Europe and North America [20]. Together, these findings indicate not only a geographic expansion but also a lengthening and intensification of transmission seasons [20]. Beyond shifts in transmission dynamics, vector-borne diseases can affect children through specific pathophysiological mechanisms that extend well beyond acute infection. Neuroinvasive manifestations are particularly relevant. For example, Lyme neuroborreliosis can involve both the central and peripheral nervous systems, leading to cranial nerve palsies, cognitive impairment, and behavioral disturbances that may interfere with school attendance and academic performance [21,22]. West Nile virus infection, although often asymptomatic, can progress to severe neuroinvasive disease in pediatric cases, including encephalitis, seizures, and acute flaccid paralysis [23]. Zika virus, in contrast, is associated with congenital infection, resulting in microcephaly, structural brain abnormalities, and long-term neurodevelopmental impairment, as well as Guillain–Barré syndrome [23]. Taken together, these diseases pose substantial risks for children, who may experience severe acute illness and long-term consequences affecting cognitive development, academic performance, and mental health [14,22,24]. The significance of these trends becomes even clearer when considering malaria. In 2023, an estimated 432,000 children under-5 died of malaria in Africa, accounting for ∼75% to 80% of all malaria-related deaths in this region, underscoring the disproportionate burden borne by early childhood [25]. With the observed incidence increase since 2015 and mortality stagnating during the past few years, there is no surprise that projections from the *World Malaria Report 2025* [26] suggest that CC could further increase malaria-related child deaths by an additional 10% to 15% in the coming decades. Rising temperatures, altered precipitation patterns, and the expansion of *Anopheles* vectors into new ecological niches (especially in highland and previously low-transmission areas) are expected to reshape the geography and seasonality of transmission, heightening the vulnerability of children who already experience the greatest morbidity and mortality from this disease [25].

#### Waterborne, foodborne, and other climate-driven infectious diseases

Beyond vector-borne pathogens, CC reshapes the risk landscape for a broader spectrum of infectious diseases through its effects on water, food systems, and extreme weather.

Extreme heat and prolonged droughts, both increasingly frequent in the CC context, alter water quantity and quality, influencing survival and growth of pathogens and increasing the risk of diarrheal diseases [14,27]. An analysis of 51 LMIC reported that the incidence of diarrhea in children under-5 increased by 5% during mild 6-mo droughts and by 8% during severe droughts [27]. If the mean global temperature exceeds 2°C increase since the Industrial Revolution, annual deaths attributable to temperature-sensitive enteric infections could surpass 10,000 by 2050 to 2065 [28]. Soil health plays a crucial role in maintaining stable and healthy conditions; soils with high organic matter percentage, good and stable structure, high biodiversity improve water infiltration, reduce pathogen transport, and limit fecal contamination of water sources. Conversely, soil degradation amplifies climate-related risks of waterborne and foodborne infections, highlighting soil stewardship as a preventive public health agroecological intervention [29,30].

Floods, tropical storms, and cyclones compromise health and environmental hygiene by damaging water, sanitation and hygiene infrastructures, mobilizing sediment contaminated with fecal pathogens, and destroying health facilities [14,28]. These events are strongly associated with outbreaks of cholera, rotavirus, and other foodborne and waterborne infections [6,31]. Furthermore, extreme weather events often lead to massive migration forcing communities into overcrowded refugee camps with poor sanitation, thereby amplifying health risks for children [14,16,27]. According to the UNICEF, “weather-related disasters caused 43.1 million internal displacements of children in 44 countries over a six-year period, ∼20,000 child displacements a day” [32]. Migration exposes them to unfamiliar pathogens in new environments, a risk that is exacerbated because migrant families frequently reside in impoverished areas or camps, where access to healthcare and preventive services, like vaccinations, is limited [33]. Migration often results in suboptimal vaccination coverage that is lower than the 90% to 95% proportion ensuring herd immunity, thus leaving children even more vulnerable to most vaccine-preventable communicable diseases [14]. Moreover, CC may accelerate antimicrobial resistance by increasing the prevalence of infectious diseases, which in turn drive higher antibiotic use and produce the conditions favoring selection and spread of drug-resistant mutants [30].

#### Knowledge gaps

Although the mechanisms linking CC to infectious disease risks in children are increasingly well characterized, significant knowledge gaps remain. Longitudinal, pediatric-specific, and geographically granular studies are largely absent: a scoping review of the literature identified a clear lack of longitudinal research and noted that most evidence still derives from high-income countries (HICs), with low-income settings and tropical regions systematically underrepresented [6]. This geographic bias has been confirmed by a systematic review of empirical research on climate and infectious diseases, which found that the majority of studies were conducted in temperate, HICs, neglecting directly transmitted diseases in tropical [34]. This pattern is particularly evident in sub-Saharan Africa, where diseases beyond malaria, including diarrheal illnesses and arboviruses, remain poorly characterized in the empirical literature, with most evidence derived from modeling studies rather than field data [35]. Across all settings, outcomes are rarely disaggregated by age subgroup within the pediatric population: a systematic evidence gap map covering a decade of research confirmed limited studies on vector-borne diseases in children and a striking underrepresentation of health outcomes for those aged 5 to 18 y, hindering the design of life-stage-appropriate adaptation strategies [36].

### CC and NCDs

NCDs have multifactorial causes, ranging from genetics, metabolic syndrome, overweight/obesity, mental stress, infections, unhealthy lifestyle, socioeconomic inequalities to environmental and climate exposure [[37], [38], [39], [40]]. The climate crisis may be particularly harmful when faced prenatally and during childhood, as physiological changes and increased biological plasticity during these periods make developing organs and systems vulnerable to biological and psychosocial threats.

An increasing body of epidemiological evidence documents associations between climate-related exposures during the intrauterine period and adverse pregnancy and birth outcomes, including preterm birth, stillbirth, and low birth weight (Table 1, references [6,8,10,11,[41], [42], [43], [44], [45], [46], [47], [48], [49], [50], [51], [52], [53], [54], [55], [56], [57], [58], [59], [60], [61], [62], [63], [64], [65], [66]]). These adverse outcomes are themselves recognized as risk factors for the development of NCDs in offspring [8,11,61,[67], [68], [69], [70]]. Additionally, climate-related exposures have been linked to poor health outcomes in the pediatric population, such as chronic respiratory diseases, allergic disorders, mental disorders, cardiovascular conditions (Table 1, references). Although the exact biological mechanisms through which CC influences the aforementioned adverse health effects are not fully understood yet, several plausible mechanisms, including oxidative stress, systemic inflammation, and immune and endocrine dysregulation may account for these observations [71,72].

**TABLE 1:** Associations of climate risks’ exposure during critical developmental stages (pregnancy, childhood, and adolescence) with adverse prenatal and pediatric health outcomes.

Population group	Climate-related event	Health outcome
Pregnant women	Heat	Hypertensive disorders and gestational diabetes mellitus [8,[41], [42], [43], [44], [45]]. Congenital anomalies [41,45]. Preterm birth, miscarriage, stillbirth, small for gestational age, and low birth weight [[41], [42], [43], [44], [45]].
	Wildfire	Preterm birth and low birth weight [43,[46], [47], [48]].
	Flood	Pregnancy loss [49]. Eclampsia/pre-eclampsia [43]. Preterm births, stillbirth, and small for gestational age [50].
	Hurricanes	Preterm births and low birth weight [43,51].
	Extreme weather events	Posttraumatic stress disorder, mood and anxiety disorders, and depression [52,53].
Youth	Storms, floods, and wildfires	Mental disorders such as stress, posttraumatic stress disorder, depression, and anxiety [[54], [55], [56]]. Cognitive deficit and learning problems [[54], [55], [56]].
	Nonoptimal temperature (both cold and heat)	Global mortality rate of cardiovascular diseases of 0.11/100,000 population, with a corresponding disability-adjusted life years rate of 8.91/100,000; burden from low temperature higher than from high temperature [57].
	Heat/high temperature	Increased pediatric emergency department visits or hospital admissions [58,59]. Heatstroke, electrolyte imbalance, dehydration, kidney diseases, and respiratory diseases [6,10,44]. Slowed cognitive and emotional functioning [11,[60], [61], [62]]. Schizophrenia, depression, composite mental health illnesses, and suicide [59].
	Heat, wildfires, thunderstorms, floods, dust storms, and climate change-related aeroallergens	Exacerbation of air pollution-related adverse health outcomes: - Allergic disorders and chronic respiratory diseases [11,44,[63], [64], [65]]. - Neurodevelopmental and cognitive impairment [44,61,66].

During pregnancy, exposure to high temperatures can induce maternal heat stress, triggering inflammatory and oxidative responses as well as dehydration, which may impair placental function and reduce uteroplacental blood flow, limiting fetal oxygen and nutrient supply and increasing the risk of adverse birth outcomes [44,73]. Heat exposure may activate the maternal hypothalamic–pituitary–adrenal (HPA) axis, disrupting endocrine and immune homeostasis, potentially affecting fetal growth trajectories and metabolic programming [44,73]. In addition, CC contributes to worsening air quality by increasing ozone, particulate matter, and wildfire-related pollutants [44], which can induce oxidative stress, systemic inflammation, endocrine disruption, epigenetic modifications, and placental dysfunction, compromising fetal development and birth outcomes [74]. Some pollutants can cross the placenta, potentially impairing fetal immune and respiratory development and increasing susceptibility to childhood asthma and allergic diseases [75]. Extreme weather events disrupt ecosystems and increase exposure to environmental toxicants, adversely affecting birth outcomes and interfering with fetal endocrine, neurological, and metabolic development [75]. Climate-related social disruptions (i.e., displacement, food system damages, and socioeconomic instability) may also increase maternal psychosocial stress and HPA-axis activation, with downstream effects on fetal neurodevelopment [76]. Climate-related food insecurity may compromise maternal nutrition, contributing to fetal growth restriction and long-term metabolic programming via epigenetic mechanisms [77].

In children and adolescents, rising temperatures and CC-related air quality deterioration can promote oxidative stress, airway inflammation, and impaired lung function, increasing the risk of respiratory morbidity [10,78], whereas acute events such as thunderstorm-related asthma and dust storms may exacerbate these conditions [79]. Elevated temperatures and atmospheric carbon dioxide (CO_2_) levels are also associated with increased aeroallergen production and prolonged pollen seasons, contributing to allergic diseases [10,44,80]. Pediatric populations are particularly vulnerable to temperature extremes due to immature thermoregulatory systems, with cold representing the leading contributor to cardiovascular disease (CVD) burden [57]. Heat exposure can lead to vasodilation, sweating, and tachycardia, increasing the likelihood of cardiovascular events [44,57], whereas cold exposure promotes blood viscosity, thrombogenesis, and vasoconstriction, elevating blood pressure and cardiac workload [57]. Moreover, extreme weather events and related environmental degradation and displacement can increase psychosocial stress and exposure to toxicants, potentially impairing children’s neurodevelopment and mental health through mechanisms involving HPA-axis dysregulation [81]. Awareness of CC as a global threat may further contribute to psychological distress among young populations [44]. Finally, evidence from a systematic review and meta-analysis indicates that climate-related exposures, e.g., droughts, floods, and long-term fluctuations in temperature and precipitation, are significantly associated with undernutrition in children, with drought linked to nearly a 50% increase in the odds of wasting and underweight [82].

Overall, CC may act as an early-life amplifier of risk factors for pediatric NCDs. Even if CC does not necessarily cause NCDs, it still constitutes a significant additional stressor, worsening existing risk factors or exacerbating the symptoms of pre-existing morbidities, through a combination of environmental influences and biological changes within families, communities, and social contexts [55]. This is concerning given the projected escalation of climate-related risks for children [3] and the already substantial global burden of pediatric NCDs. An estimated 2.1 billion children and adolescents are affected or exposed to their risk factors [83], with NCDs accounting for 45% of the 152 million disability-adjusted life years lost among those aged 5 to 14 y in 2023 [84], and 28.6% of deaths among individuals aged 5 to 19 y in 2019 [85].

Furthermore, efforts are needed to address knowledge gaps in the links between CC and pediatric NCDs. Priorities include advancing integrated, multidisciplinary research that combines climate science and health data; strengthening evidence on the short- and long-term impacts of climate-related hazards; and better characterizing the contextual and socioeconomic factors that shape vulnerability and health outcomes [6,9,11].

## Food Insecurity as a Pathway Linking CC and Pediatric Health

### Global trends in food and nutrition insecurity and pediatric health outcomes

Food and nutrition security exists when all people, at all times, have physical, social and economic access to sufficient, safe and nutritious food that meets their dietary needs and food preferences for an active and healthy life [86]. It encompasses 6 dimensions (Table 2): food availability, food access, food utilization, stability, sustainability, and agency [86].

**TABLE 2:** The description of the 6 dimensions of Food and Nutrition Security.

Dimension and attributes
*Food availability* refers to the presence of sufficient, safe, nutritious, diverse, and culturally appropriate food. This relies on agricultural production, food stocks, and trade systems.
*Food access* encompasses physical, economic, and social access to food. It depends on the location/proximity of food markets or infrastructures, purchasing power (household income), and food prices.
*Food utilization* refers to the body’s ability to absorb and use nutrients to meet its needs, influenced by feeding practices, food preparation techniques, dietary diversity, and individual health status.
*Stability* relates to the firmness over time of the aforementioned dimensions. It reflects resilience to shocks such as climate hazards and stressors, conflicts, economic downturns, or health crises like pandemics.
*Sustainability* implies the capacity of food systems to ensure food and nutrition security over the long term, without depleting environmental, social, or economic resources, thereby guaranteeing intergenerational equity and well-being.
*Agency* involves the ability/empowerment of individuals and communities to make their own choices about food, including meaningful participation in shaping food-related systems, policies, and governance.

Insecurity arises when any of these dimensions are compromised [86]. Inadequate and inequitable access to safe, sufficient, and nutritious food constitutes a violation of the right to food [87]. In 2024, an estimated 2.3 billion people (28% of the global population) were moderately or severely food insecure [88], including millions of children aged <15 y [89]. Moderate food insecurity refers to the inability to regularly eat healthy, balanced diets due to resource constraints, resulting in reduced dietary quality and increased risk of micronutrient deficiencies and other diet-related issues [88]. Severe food insecurity involves disrupted eating patterns, including running out of food, experiencing hunger, and, in extreme cases, prolonged food deprivation, seriously harming individuals’ health and well-being [88]. In addition, an estimated 638 to 720 million people (8.3% of the global population) faced hunger, or chronic undernourishment, defined as a habitual insufficiency in dietary energy intake required to sustain a normal, active, and healthy life [88].

According to *the Sustainable Development Goals (SDG) Report 2025*, although global hunger and food insecurity have declined in recent years, they remain above pre-pandemic levels [90]. Progress toward SDG2 (ending hunger, achieving food security and improved nutrition, and promoting sustainable agriculture) remains off track. This is evident for targets 2.1 (ensuring year-round access to safe, nutritious, and sufficient food) and 2.2 (ending all forms of malnutrition). The term malnutrition encompasses 2 groups of conditions [91]: one is undernutrition, which includes stunting (low height-for-age), wasting (low weight-for-height), underweight (low weight-for-age); the other is overweight and obesity (high weight-for-height) as well as diet-related NCDs, such as heart disease, stroke, diabetes, and cancer. It also includes micronutrient deficiencies or insufficiencies (a lack of vitamins and minerals) [91]. Globally, 150.2 million (23.2%) of children under-5 are stunted, 42.8 million (6.6%) are wasted, and 35.5 million (5.5%) live with obesity [92]. SDG2 is devoted to food and its related challenges, as well as promoting sustainable agriculture [93]. The definition of the SDG2, its targets 2.1 and 2.2, and the related indicators are shown in Table 3. Such a burden prevalently falls on LMICs and social-economically disadvantaged households or population groups, aggravating the existing inequalities [90]. Pregnant women, infants, and children are the most vulnerable, due to their unique physiology and metabolism combined with socioeconomic and political marginalization [7]. In pediatric populations, for example, food insecurity is associated with stunting, underweight, anemia, impaired cognitive development, and mental health issues, and in some context coexists with obesity due to reliance on low-cost, nutrient-poor foods [94].

**TABLE 3:** The SDG 2, its targets 2.1 and 2.2, and the related indicators.

SDG 2	Targets		Indicators	
End hunger, achieve food security and improved nutrition, and promote sustainable agriculture	2.1	Universal access to safe and nutritious food—by 2030, end hunger and ensure access by all people, in particular the poor and people in vulnerable situations, including infants, to safe, nutritious and sufficient food all year round	2.1.1	Prevalence of undernourishment
			2.1.2	Prevalence of moderate or severe food insecurity in the population, based on the food insecurity experience scale
	2.2.	End all forms of malnutrition—by 2030, end all forms of malnutrition, including achieving, by 2025, the internationally agreed targets on stunting and wasting in children aged <5 y, and address the nutritional needs of adolescent girls, pregnant and lactating women and older persons	2.2.1	Prevalence of stunting (height-for-age <−2 SD from the median of the WHO child growth standards) among children aged <5 y
			2.2.2	Prevalence of malnutrition (weight-for-height >+2 or <−2 SD from the median of the WHO child growth standards) among children aged <5 y, by type (wasting and overweight)

Abbreviation: SDG, sustainable development goal.

Early-life nutrition is critical, as inadequate diets during pregnancy and childhood may have long-term consequences and perpetuate intergenerational cycles of poor health [86,95]. In early childhood (0–23 mo), food insecurity can disrupt optimal feeding practices, including exclusive and continued breastfeeding [96]. Exclusive breastfeeding provides safe, accessible, and nutritionally adequate nourishment, supporting growth, immunity, and long-term health [97], and maintaining hydration in hot conditions [98]. The WHO and UNICEF recommend starting within 1 h of birth, continuing exclusively for 6 mo, then with appropriate complementary foods up to 2 y or beyond [99]. Yet globally, fewer than half of infants aged <6 mo are exclusively breastfed [88]. In addition, only 34% of children aged 6 to 23 mo meet minimum dietary diversity, defined as consumption of ≥5 of 8 key food groups [88]. As a result, many complementary diets lack essential nutrients, such as iron, zinc, calcium, and vitamin A [86]. Poor-quality, unsafe, and monotonous early diets can increase susceptibility to infectious and reinforce a “vicious cycle” of undernutrition, impairing growth, neurodevelopment, immune and endocrine function, predisposing individuals to the risk of obesity and NCDs [14,95,100,101]. Furthermore, children and adolescents in food-insecure households often face both insufficient food intake and poor diet quality, or overconsume cheap, energy-dense foods high in fats, refined sugars, and sodium, while consuming fewer fruits, vegetables, and fiber, further worsening health outcomes [86,88,102].

### Climate-induced disruptions to food systems and pathways to child malnutrition

CC is a major driver of food and nutrition insecurity, interacting with and amplifying other stressors such as unsustainable food systems, political instability, conflicts, pandemics, economic challenges (e.g., unemployment, rising food prices, poverty), and rapid urbanization [86,88]. Climate-related shocks, including extreme weather events, rising temperatures, and ecosystem degradation, pose significant challenges to food and nutrition security by impacting all components and actors within food systems. These include the production, processing, distribution, and consumption of agricultural, forestry, fisheries, and food industry products, as well as broader economic, societal, political, and natural environments. Such impacts have important consequences for all dimensions of sustainable development [[103], [104], [105]]. Table 4 [[106], [107], [108], [109], [110], [111], [112], [113]] summarizes the findings of studies investigating the impact of climate-related events on food and nutrition security. Extreme weather events and rising temperatures lead to reduced crop yields and livestock productivity. They also alter the patterns of pests and pathogens that threaten crops and animals [114], cause the loss of pollinators and biodiversity [106,115], and damage critical food system infrastructures, constraining food availability and stability. In 2024, weather extremes were the leading driver of food crises in 18 countries, affecting more than 96 million people with acute food insecurity [116]. Acute food insecurity refers to food insecurity in a specific area at a given point in time, at a severity that threatens lives and/or livelihoods [88,90].

**TABLE 4:** The impact of climate-related events on food and nutrition security.

Climate change proxy	Outcome
Extreme weather events and temperatures	In 2022, Western Europe experienced a decline of 30% in wheat and rice yields [106]. Over the past decade, in India, rice yields dropped by 10%–40%, chickpea yields by 45%, and milk production from cattle and buffalo has decreased by ≤50% [107]. Globally, over the last 30 y, annual losses have amounted to ∼69 million tons of cereals, 40 million tons of fruits, vegetables, and sugar crops, and around 16 million tons of meat, dairy products, and eggs. These losses have resulted in a food energy deficit of about 147 kcal per person per day [106]. Sub-Saharan Africa, the Near East and North Africa, as well as Latin America and the Caribbean, have experienced the highest losses in total agricultural productivity growth attributable to climate change since 1961 [108].
Elevated levels of CO_2_	Modeling studies indicate that by 2050, the anticipated rise in CO_2_ emissions could: • Reduce the global availability of protein by 19.5%, zinc by 14%, iron by 13.6% [109]. • Lower the global availability of vitamin B complex by 12.7%–30.3% [110]. • Lead to an additional 175 million, 122 million, and 132 million people suffering from zinc, protein, and folate deficiency, respectively; increase the risk of anemia among children under-5 and women of childbearing age [111,112].
Warming	By the year 2100, projections indicate that under a “no mitigation” scenario, the availability of calcium from fisheries is forecast to decline by 41%, that of iron by 37%, and that of ω-3 fatty acids and protein by 27% and 22%, respectively [113].

Abbreviation: CO_2_, carbon dioxide.

Elevated levels of CO_2_ have been shown to decrease protein concentrations and micronutrient content in staple crops such as rice, wheat, potatoes, and barley [[109], [110], [111], [112],[117], [118], [119]]. The nutrient loss driven by CO_2_ represents a serious threat to global nutritional sufficiency, potentially worsening an already critical situation where ∼2 billion people, including 372 million preschool-aged children and 1.2 billion women of reproductive age, are micronutrient deficient [120]. This is concerning given that the population is projected to grow by 2 billion by 2050 [88].

CC also threatens aquatic food systems, reducing the potential for fish catches and lowering the seafood nutritional quality [113,121]. Ocean warming, acidification, deoxygenation, and declining phytoplankton biomass impair fish survival, growth, reproduction, and biodiversity [113,121]. Heat-driven plankton declines, which form the basis of the marine food web, can hinder omega (ω)-3 fatty acid production and transfer, reducing their concentration in fish [122]. These impacts limit the provision of essential nutrients such as iron, calcium, protein, and ω-3 fatty acids, with implications for human nutrition and health [113,122].

Climate shocks further constrain food access through rising prices, income loss, and reduced purchasing power, driving dietary shifts from diverse, nutrient-rich diets toward cheaper, energy-dense, nutrient-poor foods [88]. A 10% rise in food prices is linked to a 3.5% increase in moderate or severe food insecurity and a 2.7% to 4.3% increase in wasting among children under-5 [88]. In South Africa, severe droughts were associated with significant changes in children’s nutritional status, with the largest increases in stunting and obesity, likely due to reduced access to nutritious foods and higher food prices [123]. Long-term warming is also associated with lower dietary diversity in children [124].

CC additionally affects nutrient utilization: floods, droughts, and damaged infrastructure increase exposure to contaminated food and water, raising diarrheal diseases that impair nutrient absorption, whereas climate-sensitive illnesses like malaria and dengue both exacerbate and are worsened by malnutrition [125].

Food insecurity thus emerges as a key pathway linking CC to malnutrition and poor health, particularly in children, through insufficient food quantity and/or poor dietary quality. Diets dominated by starchy staples and low diversity often lack adequate energy, high-quality protein, and essential micronutrients, leading to undernutrition, micronutrient deficiencies, impaired growth, and weakening immunity, increasing susceptibility to infectious diseases and long-term risk of NCDs. Simultaneously, reliance on low-quality, energy-dense diets with limited fruits and vegetables can promote systemic inflammation and abdominal obesity, increasing the risk of overweight, obesity, and NCDs [86].

CC, together with undernutrition and obesity, constitutes the “global syndemic,” a set of interacting epidemics driven by shared systemic factors such as urbanization, motorized transport, and the globalization of unhealthy diets [126].

## Revisiting Food Environments for Climate and Health

The relationship between CC and eco-agrofood systems is not unidirectional [95]: CC causes harm to the agro-ecosystem; conversely, the food supply chain, from farm to fork, acts as a driver of CC, imperiling the planet ecosystem stability. Today’s eco-agrofood systems and patterns of consumption, based on energy-dense, animal, and processed foods, exert significant environmental pressures in terms of greenhouse gases emissions and ecosystems degradation (e.g., soil degradation, loss of biodiversity, and water scarcity), with animal-based foods being responsible for the highest impacts [127,128]. Concurrently, these patterns, which are low in fiber and rich in salt, sugars, and saturated fats, contribute to the global burden of mortality and morbidity, thus representing a common determinant for both human and planetary health. Furthermore, urbanization has contributed to increased inequalities in food access, leading to the creation of food deserts and swamps in poor neighborhoods [129]. Food deserts are prevalent worldwide: across countries and regions, they disproportionately affect high-poverty communities [129]. These areas often lack access to fresh, nutritious foods like fruits, vegetables, and whole grains, while being surrounded by outlets that sell mostly low-quality, cheaper fast foods, sugary drinks, and snacks. Economic barriers and structural factors can make it difficult for families to afford healthier options and animal-source proteins, which tend to be more expensive and time-consuming to prepare [88]. Consequently, children and adolescents in these communities may consume more sugary beverages and energy-dense, nutrient-poor foods, which can contribute to health issues such as obesity, diabetes, and dental problems [100,[130], [131], [132]].

Hence, the urgency of addressing the crucial structural determinants of health and shift toward healthy, sustainable diets and food systems to achieving the SDGs and the Paris Agreement [133], with the EAT_Lancet Commission Planetary Health Diet (PHD) serving as a foundational, globally adaptable framework that respects cultural and regional diversity [87]. The PHD prioritizes diverse, whole or minimally processed, primarily plant-based foods (i.e., whole grains, fruits, vegetables, nuts, and legumes), alongside moderate consumption of fish, dairy, and meat. It also advocates for mostly unsaturated fats, excludes partially hydrogenated oils, and limits added sugars and salt.

Tackling effectively the interconnected challenges of CC, food insecurity, pediatric malnutrition, and related morbidities, including both NCDs and infectious diseases, require an urgent scaling up double- and triple-duty actions, i.e., integrated intervention, policies, and programs that simultaneously address 2 or more components of the global syndemic [126,[134], [135], [136]]. The main characteristics of these integrated actions and policies are summarized in Table 5 [7,33,86,126,128,[134], [135], [136], [137], [138], [139], [140]]. Adopting a “glocal” approach—an integrated strategic approach that takes into account both global and local contexts (the term “glocal” has been coined by Kickbusch to indicate such approach [141])—is also essential [142], recognizing that while CC and related challenges are a global phenomenon requiring international coordination, targets and interventions must be diversified to reflect and respect the specificities of local populations, e.g., regional disease burdens, environmental challenges, and cultural traditions [87,128,136,143].

**TABLE 5:** The main characteristics of integrated actions and policies that address the interconnected challenges of climate change, food insecurity, pediatric malnutrition, and related morbidities.

Key feature	Reference
Be grounded in tailored, interdisciplinary, and multisectoral strategies, endorsed by all relevant ministries and coordinated across individual, community, national, regional, and global levels.	[[134], [135], [136]]
Involve a wide range of stakeholders, including government agencies, the agrofood sector, intergovernmental organizations, educational institutions, civil society, consumers, research institutions, and public health organizations, to act collectively to advance global sustainability, health, and equity.	[137,138]
Promote behavior change while delivering multiple cobenefits, such as climate change mitigation, enhanced food and nutrition security, disease prevention, and securing social foundations for a just food system	[134]
Integrate early-life nutrition, sustainable food environments, resilient health systems, strengthened immunization programs, transformative water, sanitation and hygiene, women’s empowerment as well as urban design, geopolitical factors, and socioeconomic factors.	[7,33,86,126,134,139,140]
Educate, incentivize, and enable individuals to make informed food choices that are both health-promoting and environmentally sustainable.	[128]
Prioritize the specific needs of women of reproductive age, children, and adolescents in programs and research agendas to ensure equitable and sustainable intergenerational impacts.	[7,138]

### Strategies for transitioning to healthy, sustainable eating

#### Strategies targeting the eco-agrofood systems

Transforming eco-agrofood systems is crucial for mitigating global warming, promoting planetary sustainability as well as prioritizing diet quality, enhancing human health, and addressing socioeconomic inequalities, thus upholding human rights related to food systems, such as the rights to food, a healthy environment, and decent work [87]. To achieve this, climate adaptation and mitigation measures in agriculture, livestock, and fisheries should be implemented, diversifying production systems and integrating circular economy strategies, such as recycling across both LMICs and HICs [87,93,144]. Agroecological approach prioritizing soil health, crop diversification, and ecosystem functioning is recognized as key strategy to enhance food availability, dietary diversity, and nutrition quality, and at the same time strengthening the resilience of agrofood systems to CC [134]. The abandonment of minor and traditional crops, which typically require minimal agronomic inputs (e.g., agrochemicals, water, and tillage), together with the decline of leguminous plants, which are essential to crop rotation and soil fertility, has driven a shift toward monocropping and high-input farming systems [93]. Intensified farming systems include the misuse and overuse of antimicrobials in livestock and crops, which further drives antimicrobial multidrug resistance [30]. Some effective approaches include crop diversification, crop or grazing rotation, water-saving techniques, and recycling biodegradable food waste. These practices align with agroecological principles and underscore the central role of soil health in offering cobenefits for climate mitigation, biodiversity regeneration, resilience in pest and disease, improve yield, food security, and human health [93,143,[145], [146], [147]].

Research into climate-resilient crop varieties, animal breeds, fish species, and forest plants is also pivotal for both adaptation and conserving biodiversity [95,145]. A prudent use of antibiotics across human health, animal health, and agricultural sectors is essential to reduce the incidence and transmission of antimicrobial resistance [30].

Strengthening local and regional supply chains by promoting traditional climate-resilient cereals, fruits, and vegetables or biofortified crops can help mitigate food shortages and counteract the nutrient dilution due to CC, thus minimizing the global malnutrition challenges [136,145,148]. Agroecological systems offer a systematic response able to mitigate CC, reduce malnutrition in all its forms, and improve long-term pediatric health outcomes by restoring soil health and local food environment. Thus, agroecology directly addresses the shared drivers of this global syndemic by promoting diversified, minimal processed diets, reducing environmental degradation, and improving access to healthy food, particularly in marginalized communities.

Supporting the use of local and seasonal ingredients not only reinforces local food systems, contributing to food security by reducing dependency on global supply chains (although more than 50,000 plant species are potentially suitable for food production, only 15–20 are important on a global scale, and ∼85% of global food requirements rely on only 8 crops) [93], but also reduces long-distance transport–related energy costs and emissions. Implementing alternative food initiatives, which shorten food supply chains and localize food production and consumption, can potentially tackle food inequalities, transforming food deserts into food oases for healthy, local, and sustainable products [149]. To this aim, policymakers should assist small-scale farmers and small-to-medium-sized enterprises through subsidy programs and knowledge sharing [88,135].

The food industry should implement eco-efficient production practices that optimize resource utilization through recycling, reusing by-products, and employing sustainable packaging [87,150]; invest in developing innovative plant-based products; and increase transparency in food labeling, such as clear nutritional information and sustainability indicators.

#### Strategies targeting the consumers

To effectively raise consumer awareness and empower individuals to make informed, health-conscious, and environmentally responsible food choice, including reducing food waste, several key measures should be implemented [87,128,150]. These include: social protection interventions, such as school meals and unconditional cash transfers; integrating sustainability principles into national food-based dietary guidelines; incorporating sustainability education into school curricula, and reforming public food procurement practices to reach a wider audience. Organizing public events and media campaigns can highlight the benefits of sustainable eating. Using clear, easy-to-understand labels on food products, such as indicators for organic, locally sourced, or low-impact farming methods, further supports informed consumer decisions.

The key recommendations for transforming eco-agrofood systems and consumer behavior are summarized in Table 6.

**TABLE 6:** Key recommendations for transforming eco-agrofood systems and consumer behavior.

Target area	Key feature
Agrofood system	Integrate soil health as a cross-sector priority linking agriculture, nutrition, climate, and public health policies.
	Promote agroecological practices (e.g., crop diversification, rotations, and organic matter restoration) to enhance soil fertility, resilience, and food quality.
	Strengthen local food systems based on healthy soils to improve dietary diversity and reduce food insecurity.
	Recognize soil stewardship as a preventive health strategy, reducing pathogen transmission and supporting safe water and food systems.
Consumer	Expand social protection interventions (e.g., school meals and cash transfers) to support equitable access to healthy and sustainable diets.
	Integrate sustainability principles into national food-based dietary guidelines to promote environmentally responsible eating habits.
	Reform public food procurement practices, incorporate sustainability education into school curricula to build early awareness of sustainable eating practices.
	Introduce clear, easy-to-understand food labeling (e.g., organic, local, and low-impact) to enable informed consumer choices.

### Promoting sustainable nutrition from an early age

CC solutions that benefit children’s health should also focus on fostering more sustainable eating habits from birth, as childhood is a critical period during which basics for future dietary patterns are established [151]. The following points must be reinforced:

• *Breastfeeding.* Protect, promote, and support exclusive breastfeeding while discouraging water or other fluid supplementation and counteracting the marketing of breast milk substitutes [151]. Both health and the environment benefit, as exclusive breastfeeding is an important CC mitigation measure [152].Rationale: evidence shows that exclusive breastfeeding reduces infant mortality, infectious diseases, and later risk of obesity and chronic diseases [97]. Life-cycle assessments indicate that exclusive breastfeeding has significantly lower environmental impacts compared with formula feeding [152]. For example, compared with 4 mo of formula feeding, exclusive breastfeeding results in a 38% lower global warming potential, 72% lower terrestrial acidification, 35% lower freshwater eutrophication, 59% lower marine eutrophication, and 53% lower land use [153]. Extending exclusive breastfeeding to 6 mo reduces the carbon footprint by 48%. Additionally, a 2009 study reported that in the United States alone, every year, over 32 million kW of energy is used in processing, packaging, and transporting infant formula, and large quantities of packaging materials (i.e., 550 million cans, 86,000 tons of metal, and 364,000 kg of wrapping paper) end up in landfills [154]. Thus, breastfeeding is both biologically optimal for infants and significantly sustainable for the environment.

• *Complementary feeding.* During the complementary feeding period (6–23 mo), offer a variety of foods, flavors, and textures, to meet the high energy and nutrient demands of growth and development, gradually transitioning to finger foods and family meals [151]. Avoid foods high in free sugars, salt, and trans fats, as well as sugar-sweetened beverages [151,155]. Prioritize seasonal, local available, minimally processed foods [151].Rationale: from the health perspective, dietary diversity supports healthy growth trajectories, micronutrient adequacy, and cognitive development [155]. From a sustainability standpoint, minimally processed, locally available foods have lower environmental impacts [87]. Animal-source foods (e.g., eggs, dairy, and meat) should not be excluded in this age group, as they provide essential nutrients with minimal environmental burden at this age [151,152,155]. A balanced approach is needed: promoting diverse, sustainable eating patterns that include appropriate amounts of animal-source foods to meet nutritional needs.

• *Nutrition for ages 2 and up.* For children aged ≥2 y, as well as adolescents, encourage balanced and diverse diets, rich in whole grains, nuts, and plant-based foods, with modest consumption of animal products and minimal intake of red and processed meats, sugars, and refined grains, in line with current dietary guidelines [151]. Such diets promote health while reducing the carbon footprint [87].Rationale: evidence indicates that these dietary patterns support growth and development, and reduce the risk of NCDs [87]. For example, a prospective study reported that greater adherence to the PHD is associated with lower cardiometabolic biomarkers in adolescents, underscoring its potential to prevent the onset of CVDs [156]. Environmentally, modeling diets for Norwegian 2y-olds on the PHD demonstrated reductions in global warming potential by 37%, freshwater eutrophication by 38%, terrestrial acidification by 59%, and water use by 56%, compared with typical diets higher in red meat, dairy, and sweets [157]. Traditional healthy diets, such as the Japanese Diet, Mediterranean Diet, Nordic Diet, and diets from Indonesia, Mexico, India, China, and West Africa, already reflect the principles of sustainable and healthy eating [133]. The EAT_Lancet Commission recently emphasized the importance of cultural contexts, advocating for culturally appropriate, sustainable dietary traditions as a key factor in successfully implementing the PHD [87]. Although limiting meats and animal-source foods is important for environmental goals, these foods provide essential fatty acids, micronutrients, minerals, and vitamins, which are relevant for vulnerable populations with increased nutritional demands and/or in contexts with limited access to nutrient-rich foods [151,152]. In such cases, micronutrient supplements or fortified foods are recommended to ensure nutrient adequacy. Together, these findings highlight that during late childhood and adolescence, dietary patterns should balance health promotion, environmental sustainability, and nutritional adequacy.

To promote healthy, sustainable eating habits from an early age, a range of coordinated actions is needed. Improving breastfeeding practices requires supportive policies such as paid maternity leave, along with stricter regulation of misleading nutrition and health claims in formula marketing [152]. Enhancing access to nutritious, sustainable foods involves strengthening social protection systems (i.e., school meals, food aid, and cash transfers), expanding community-based safety nets and local markets [87,150]. Robust regulations are essential to protect children and adolescents from the aggressive marketing of unhealthy foods and beverages, which can significantly increase the appeal of junk food [158].

Urgent implementation of sustainable, age-specific, and context-relevant dietary recommendations is needed to address persistent gaps in defining optimal intakes across pediatric populations and settings [151].

Health professionals, particularly pediatricians, also play a key role by educating families and communities about nutritious, climate-friendly lifestyles and offering practical guidance [80,159,160], mostly in settings where caregivers lack adequate awareness of nutrition, hygiene, or sustainability. Collectively, these strategies can shape healthier food environments for infants, children, and adolescents, and provide long-term well-being for individuals and the planet.

In conclusion, as of 2026, children are facing a confluence of interconnected global pandemics, namely CC, food insecurity, malnutrition in all its form, and NCDs, centered around the CCagrofood environment dyad. Empirical evidence indicates that exposure to climate-related hazards and stressors during developmental stages, from conception through adolescence, is associated with an elevated risk of both acute and chronic diseases in the pediatric population, with potentially life-threatening and transgenerational consequences. Although the exact mechanisms by which CC triggers adverse health outcomes have not been fully explained yet, climate-driven environmental changes significantly challenge food and nutrition security by impacting the entire eco-agrofood system. This can increase the risk of pediatric malnutrition, thereby contributing to the spread of diseases in the short- and long-term. Contemporary agrofood systems are not only highly vulnerable to CC but also significantly drivers of its acceleration, creating a feedback loop that worsens health and social inequities. Current dietary patterns are often nutritionally inadequate, further increasing the global burden of morbidity and mortality. The most marginalized households and LMICs are disproportionately vulnerable and bear the greatest burdens of these global crises. Breaking the cycle linking CC, food insecurity, and diseases requires a life approach that fosters healthy, sustainable, and equitable eating habits from early life. This includes prioritizing breastfeeding in infancy, ensuring dietary diversity during complementary feeding, and promoting sustainable dietary patterns in later childhood and adolescence, accounting for cultural and socioeconomic contexts.

Addressing these intertwined crises demands a holistic framework integrating health, nutrition, environmental sustainability, and social equity. This involves a substantial transformation of agrofood systems alongside strengthening individual and collective knowledge, awareness, and empowerment, within a “glocal” framework that links global strategies with local implementation. Cross-sector partnerships and interdisciplinary actions are crucial to drive meaningful change. Agroecology and soil health should be recognized as integral components of global health strategies, given their foundational role in shaping the agrofood environment, nutrition quality, and resilience to climate-related shocks. Embedding soil-centered approaches within climate, nutrition, and health policies may represent a critical leverage point to protect pediatric health and reduce global health inequities across generation. Finally, further research targeted on pediatric populations is essential to elucidate the causal pathways linking CC and health, and to inform early-life nutrition interventions respectful of, and grounded within, the principles of global and planetary health.

## Author contributions

The authors’ responsibilities were as follows – TS, DM: collected and interpreted data, wrote the paper; CB: designed the research, collected and interpreted data, wrote the paper, hold primary responsibility for final content, and supervised the research; and all authors: review of the manuscript, read and approved the final manuscript.

## Declaration of generative AI and AI-Assisted Technologies in the Writing Process

During the preparation of this work, the authors used ChatGPT (OpenAI) in order to refine English language. After using this tool, the authors reviewed and edited the content as needed and take full responsibility for the content of the publication.

## Funding

This study was partially funded by Italian Ministry of Health, Current research IRCCS (Istituto di ricovero e Cura a Carattere Scientifico). The funders had no role in the writing of the present paper.

## Conflict of interest

CA is an Editorial Board Member for *Advances in Nutrition* and played no role in the Journal’s evaluation of the manuscript. All other authors report no conflicts of interest.
